# Explaining the striking difference in twist-stretch coupling between DNA and RNA: A comparative molecular dynamics analysis

**DOI:** 10.1093/nar/gkv1028

**Published:** 2015-10-12

**Authors:** Korbinian Liebl, Tomas Drsata, Filip Lankas, Jan Lipfert, Martin Zacharias

**Affiliations:** 1 Physik-Department T38, Technische Universität München, James-Franck-Strasse, D-85748 Garching, Germany; 2 Institute of Organic Chemistry and Biochemistry, Academy of Sciences of the Czech Republic, Flemingovo namesti 2, 166 10 Prague, Czech Republic; 3 Department of Physical and Macromolecular Chemistry, Faculty of Science, Charles University Prague, Albertov 6, 128 43 Prague, Czech Republic; 4 Laboratory of Informatics and Chemistry, University of Chemistry and Technology Prague, Technická 5, 166 28 Prague, Czech Republic; 5 Department of Physics, Center for Nanoscience (CeNS), and Nanosystems Initiative Munich (NIM), Ludwig-Maximilian-University Munich, 80799 Munich, Germany

## Abstract

Double stranded helical DNA and RNA are flexible molecules that can undergo global conformational fluctuations. Their bending, twisting and stretching deformabilities are of similar magnitude. However, recent single-molecule experiments revealed a striking qualitative difference indicating an opposite sign for the twist-stretch couplings of dsDNA and dsRNA [Lipfert *et al*. 2014. Proc. Natl. Acad. Sci. U.S.A. 111, 15408] that is not explained by existing models. Employing unconstrained Molecular Dynamics (MD) simulations we are able to reproduce the qualitatively different twist-stretch coupling for dsDNA and dsRNA in semi-quantitative agreement with experiment. Similar results are also found in simulations that include an external torque to induce over- or unwinding of DNA and RNA. Detailed analysis of the helical deformations coupled to twist indicate that the interplay of helical rise, base pair inclination and displacement from the helix axis upon twist changes are responsible for the different twist-stretch correlations. Overwinding of RNA results in more compact conformations with a narrower major groove and consequently reduced helical extension. Overwinding of DNA decreases the size of the minor groove and the resulting positive base pair inclination leads to a slender and more extended helical structure.

## INTRODUCTION

Conformational flexibility is of central importance for the recognition and function of DNA and RNA molecules (1–3). In addition, nucleic acids are being increasingly used as building materials for engineered nanostructures (4). Both double-stranded (ds)DNA and dsRNA are not rigid but can undergo deformations such as bending, twisting and stretching that lead to global changes in their conformations (5–10). The global deformability of double-stranded nucleic acids is characterized by bending, twisting, and stretching elasticities and the coupling of these parameters. Recent progress in single molecule manipulation has contributed to the accurate experimental characterization of the global deformability in particular of DNA (11–22). The elastic deformabilities are characterized by the bending persistence length (P_DNA_∼50 nm, P_RNA_∼60 nm) (23–27), the twist persistence length (C_DNA_∼109 nm, C_RNA_∼100 nm) (27–29) and the stretch or Young's modulus (26,27,30,31) (S_DNA_∼1000 pN, S_RNA_∼400 pN). These characteristic quantities can be translated into approximate mean bending fluctuation per base pair step (<θ>∼(2rise/P)^0.5^; θ_DNA_ = 6.5°; θ_RNA_ = 4.5°), thermal mean twist fluctuation per base pair step (<τ>∼(rise/(2π2C))^0.5^; τ_DNA_ = 0.67°; τ_RNA_ = 0.72°) and helical rise fluctuation (<z_DNA_>∼0.14 Å; <z_RNA_>∼0.16 Å) (32). The comparison indicates that overall the global flexibility of DNA and RNA is similar to within ∼40% as might be expected from the overall similar dimensions and composition of the two molecules. However, recent single-molecule magnetic tweezers experiments have revealed a striking quantitative and qualitative difference in the twist-stretch coupling of dsDNA and dsRNA (27): while dsDNA increases its helical extension upon over-twisting (by definition a negative twist-stretch coupling) (9,15–17,27,33,34) dsRNA exhibits the opposite behavior, namely a reduction of helical extension for overwinding (27) (experimental measurements summarized in Supplementary Information Table S1).

Intuitively, for simple models of a helical elastic polymer an increase of the twist of the helix (meaning one tries to wrap the strands more often around an embedding cylinder) requires a shortening of the duplex extension along the cylinder direction. Historically, this behavior was assumed for DNA (35,36) and deduced—incorrectly—from early single molecule studies (35–37) until more recent experimental evidence indicated the opposite behavior (15,33). Several models have been proposed to explain the negative twist-stretch coupling in DNA in terms of a stiff helical backbone wrapped around a soft isotropic code (15,38). While these models can account for the sign and magnitude of the twist-stretch coupling for DNA, they predict RNA to lengthen upon overwinding like DNA, in clear disagreement with experimental results (27).

Simulations of nucleic acids at varying levels of complexity have yielded predictions about the twist-stretch couplings. Kosikov *et al*. (39) studied DNA homopolymers employing all-atom potentials and an implicit treatment of the solvent and ion atmosphere in a molecular mechanics framework and found that A-form DNA tends to shorten while overwinding while B-form DNA lengthens, in qualitative agreement with experimental results. However, these simulations are for DNA only and the predicted magnitudes of the twist-stretch couplings are too large by factors of 3–5. A computational study employing a coarse-grained nucleic acid model for DNA and RNA based on a statistical base-pair step potential extracted from known nucleic acid structures could satisfactorily reproduce the bend, twist and stretch elasticities, but failed to account for the opposite sign of twist-stretch coupling for dsRNA and dsDNA (40). A theoretical study (41) has shown that, for helices of elastic filaments described by the classic Kirchhoff–Love model, both signs of the coupling are possible, depending on the precise elastic properties of the filament. However, the study does not make explicit predictions for possible differences in RNA and DNA. Hence, the relation between a helix of elastic filament and a double stranded DNA or RNA molecule is not straightforward. In summary, there is currently no convincing model that would explain the experimentally observed difference in the sign of the twist-stretch coupling for dsDNA and dsRNA, exposing a major gap in our current understanding of nucleic acids.

Molecular Dynamics (MD) simulations at atomic resolution and including surrounding water and ions are a useful and widely applied tool to study the dynamics and deformability of nucleic acids (8,10,42–49). For DNA systematic comparative studies on many sequence variants up to the microsecond regime have been performed resulting in conformations close to standard B-form and conformational flexibility in good agreement with available experimental data (47–49). It is also possible to reproduce global deformabilities of DNA and RNA in good agreement with experiment (9,49–52). In particular, the coupling of stretching and twisting of DNA observed in models parameterized from MD simulations agrees with experiment (9,34,52) and has been used to extract also sequence dependent effects (34). However, no comparative studies on dsDNA and dsRNA have been performed to reproduce and explain the differences in twist-stretch coupling.

Here we employed extensive comparative MD simulations (∼1 μs) on dsDNA and dsRNA molecules to investigate their differences in twist-stretch coupling. The twist-stretch coupling was analyzed directly by extracting the covariation of helical twist and helical rise from the trajectories as well as by a stiffness analysis of the MD trajectory. In addition, restraint MD simulations including a torque acting on the terminal base pairs of dsDNA or dsRNA duplexes were performed similar to the experimental torque tweezer set up (27). The analysis of unrestrained simulations as well as the restraint simulations reproduced the opposite sign of the twist-stretch couplings for dsDNA and dsRNA and magnitudes of the couplings in good agreement with experiment. Detailed analysis of the helical deformations coupled to twist indicated an interplay of helical rise, base pair inclination, and displacement from the helix axis upon twist changes as the underlying mechanism of the different twist-stretch correlations.

## MATERIALS AND METHODS

### Molecular dynamics simulations

Unrestrained MD simulations were started from regular B-form (in case of dsDNA) or A-form (for dsRNA) 16 base pair (bp) duplexes with the sequence: d(5′-GCGCAATGGAGTACGC/5′-GCGTACTCCATTGCGC) in case of DNA and r(5′-GCGCAAUGGAGUACGC/5′-GCGUACUCCAUUGCGC). The sequence contains all (10) possible dinucleotide steps at least once representing (approximately) a randomly selected sequence. The Amber12 Molecular Dynamics Package (53) was used for the simulations. All simulations were performed in explicit water (TIP3P) (54) with a rectangular box and a minimum distance of 12 Å between DNA and box boundary. Sodium counter ions were included to neutralize the system. All classical MD simulations were carried out with the pmemd.cuda module of the AMBER12 package using the parmbsc0 force field including recent additional improvements for nucleic acids (55–57). The simulation systems were first energy minimized (5000 steps) with restraints on DNA followed by 5000 steps of unrestrained energy minimization. The system was heated up in 3 stages (each stage 100 ps) to 300 K in stages of 100 K followed by gradual removal of the positional restraints from 25 kcalmol^−1^Å^−2^ to 0.5 kcalmol^−1^Å^−2^ (in 5 stages). The duplex structures were initially aligned along the long (z) axis of the box. In order to avoid overall rotation of the duplexes during simulations the nucleic acid backbone of the first two base pairs was weakly restraint to the initial placement (force constant for positional restraints: 0.1 kcalmol^−1^Å^−2^). This has little influence on the conformational fluctuations of the first base pairs (which were not used for analysis) and avoids overall rotation of the molecule. After a 20 ns equilibration simulation the MD simulations were extended to ∼1 μs data gathering time at a simulation temperature of 300 K and at constant pressure 1 bar (NPT ensemble).

In order to estimate the energetic contributions to twist/rise deformations we performed a MMGB/SA (Molecular Mechanics Generalized Born Surface Area) analysis (53) of the recorded trajectories (100 000 snapshots) after removal of explicit solvent molecules (assuming a salt concentration of 0.1 M). The total energy of a DNA/RNA conformation is given as a sum of bonded (bond length, bond angle, bond dihedral) and non-bonded (Lennard–Jones, Coulomb, Generalized Born and surface area dependent non-polar solvation) contributions. The calculated energies were ordered with respect to the mean twist/rise of the snapshots to obtain histograms of mean energies versus twist/rise.

A second set of MD simulations applied torque to the termini of the duplexes. During these simulations the first two and last two base pairs of the duplexes were weakly restraint limiting the distance in the plane perpendicular to the z-axis (representing the helical axis of the initial duplex) relative to the z-axis. This type of ‘cylindrical’ restraint allows free rotation around the z-axis and therefore full twist flexibility of the duplex. It is equivalent to a weak stretching force applied during torque tweezer experiments (27) to align the duplex along a helical axis. The force constant was 0.5 kcalmol^−1^Å^−2^. For the application of a torque on the DNA termini a pseudo dihedral angle defined by the ribose (or desxyribose) C1’ atoms of the third and the second base pair before the last base pair was defined similar to the definition used in a previous study (58). The C1’ atoms on opposite strands at both ends of the helices form a vector that is approximately perpendicular to the helical axis such that a torque causes a twist deformation. Application of a quadratic penalty potential allows adjusting the total twist of the central 12 base pairs embedded by the duplex termini during the MD simulation. In order to avoid any local distortion a weak force constant of 0.015 kcalmol^−1^deg^−2^ was used to control the overall twist of the duplexes. The torque reference was changed in steps of 2.5° which represent a mean ∼0.15° twist change per base pair step. For each restraint twist other helical variables were recorded and analyzed. The analysis of helical parameters was performed either using Curves+ (59) or 3DNA (60).

### Energy minimization using Jumna

The Jumna (junction minimization of nucleic acids) program (61) allows modeling and energy minimization of nucleic acids in terms of helical base axis coordinates (three translational variables (x-disp, y-disp and helical rise) and three rotational variables (inclination, tip and twist). In addition, the internal flexibility of each nucleotide is described by dihedral angles around the glycosidic sugar base link and the phosphodiester backbone dihedral angles. Valence angles outside the sugar ring and all bond lengths except for the connection between nucleotides are fixed at their optimum value. The constraints on the sugar ring and internucleotide connections are imposed via harmonic penalty terms. The independent variables for each nucleotide are the six helical variables, five dihedral angles and three valence angles (within the sugar ring). All other variables are dependent and are determined by the closure conditions between nucleotides (61). With Jumna it is possible to generate a nucleic acid structure in terms of helical coordinates and to lock or fix a subset of helical coordinates and to relax and to optimize the energy in terms of all other internal (and/or) helical variables.

### Analysis of DNA stiffness

In order to systematically compare the conformational flexibility of DNA and RNA, the unrestraint MD trajectories were analyzed in terms of a mechanical model with quadratic (harmonic) deformation energy. The DNA or RNA fragment is described by two global coordinates—its length *l*, defined as the sum of helical rises and its total twist *ω*, defined as the sum of helical twists of the individual steps. The deformation energy is assumed to take the form

(1) \begin{equation*} E = \frac{1}{2}\Delta {\bf w} \cdot {\bf K}\Delta {\bf w} \end{equation*}

where $\Delta {\bf w} = (\Delta w_1 ,\Delta w_2 )$ is a vector of deviations from the equilibrium coordinate values, with components

(2) \begin{equation*} \Delta w_1 = \omega - \omega _0 ,\,\Delta w_2 = l - l_0 \end{equation*}

and

(3) \begin{equation*} {\bf K} = \left( {\begin{array}{*{20}c} {k_{11} } & {k_{12} } \\ {k_{12} } & {k_{22} } \\ \end{array}} \right) \end{equation*}

is a 2-by-2 symmetric, positive definite stiffness matrix. The energy function (1) can be rewritten as

(4) \begin{equation*} 2E = \left( {k_{11} - \frac{{k_{12}^2 }}{{k_{22} }}} \right)\Delta w_1^2 + k_{22} \left( {\frac{{k_{12} }}{{k_{22} }}\Delta w_1 + \Delta w_2 } \right)^2 \end{equation*}

Notice that $k_{22} >0$, $k_{11} - k_{12}^2 /k_{22} > 0$ due to the positive definiteness of **K**. Assume that $\Delta w_1$ is constrained (fixed), while $\Delta w_2$ remains unconstrained (free). In that case $\Delta w_2$ takes such a value that the energy, consistent with the constraint, is minimal. It follows from Equation (4) that the constrained minimum is reached if and only if the expression in the second parenthesis is zero. Substituting for $\Delta w_1$ and $\Delta w_2$ from Equation (2), this condition takes the form

(5) \begin{equation*} \frac{{l - l_0 }}{{\left( {\omega - \omega _0 } \right)_{constr} }} = - \frac{{k_{12} }}{{k_{22} }} \end{equation*}

This is the desired expression of the twist-stretch coupling coefficient in terms of the elements of the stiffness matrix **K**. It describes the elongation upon imposed change of twist.

The general theory of thermodynamic fluctuations implies relations between the model parameters and moments of the canonical distribution. In particular,

(6) \begin{equation*} {\bf K} = k_B T{\bf C}^{ - 1} \end{equation*}

where

(7) \begin{equation*} {\bf C} = \left( {\begin{array}{*{20}c} {c_{11} } & {c_{12} } \\ {c_{12} } & {c_{22} } \\ \end{array}} \right) \end{equation*}

is the covariance matrix of the coordinates, *T* is the thermodynamic temperature and $k_B$ the Boltzmann constant. Details can be found in previous publications (10,44–46). Using Equations (5 and 6), the twist-stretch coupling can be expressed in terms of the elements of the covariance matrix as

(8) \begin{equation*} \frac{{l - l_0 }}{{\left( {\omega - \omega _0 } \right)_{constr} }} = \frac{{c_{12} }}{{c_{11} }} \end{equation*}

In an entirely analogous manner, we can describe the complementary case, i.e. the change of twist upon imposed elongation. The coupling coefficient is

(9) \begin{equation*} \frac{{\omega - \omega _0 }}{{\left( {l - l_0 } \right)_{constr} }} = \frac{{c_{12} }}{{c_{22} }} \end{equation*}

In our calculations, the covariances over the canonical ensemble were estimated by covariances of the coordinate time series obtained from the MD trajectories. Only the inner 10 bp of the simulated duplexes were taken into account.

## RESULTS AND DISCUSSION

**Figure 1.**
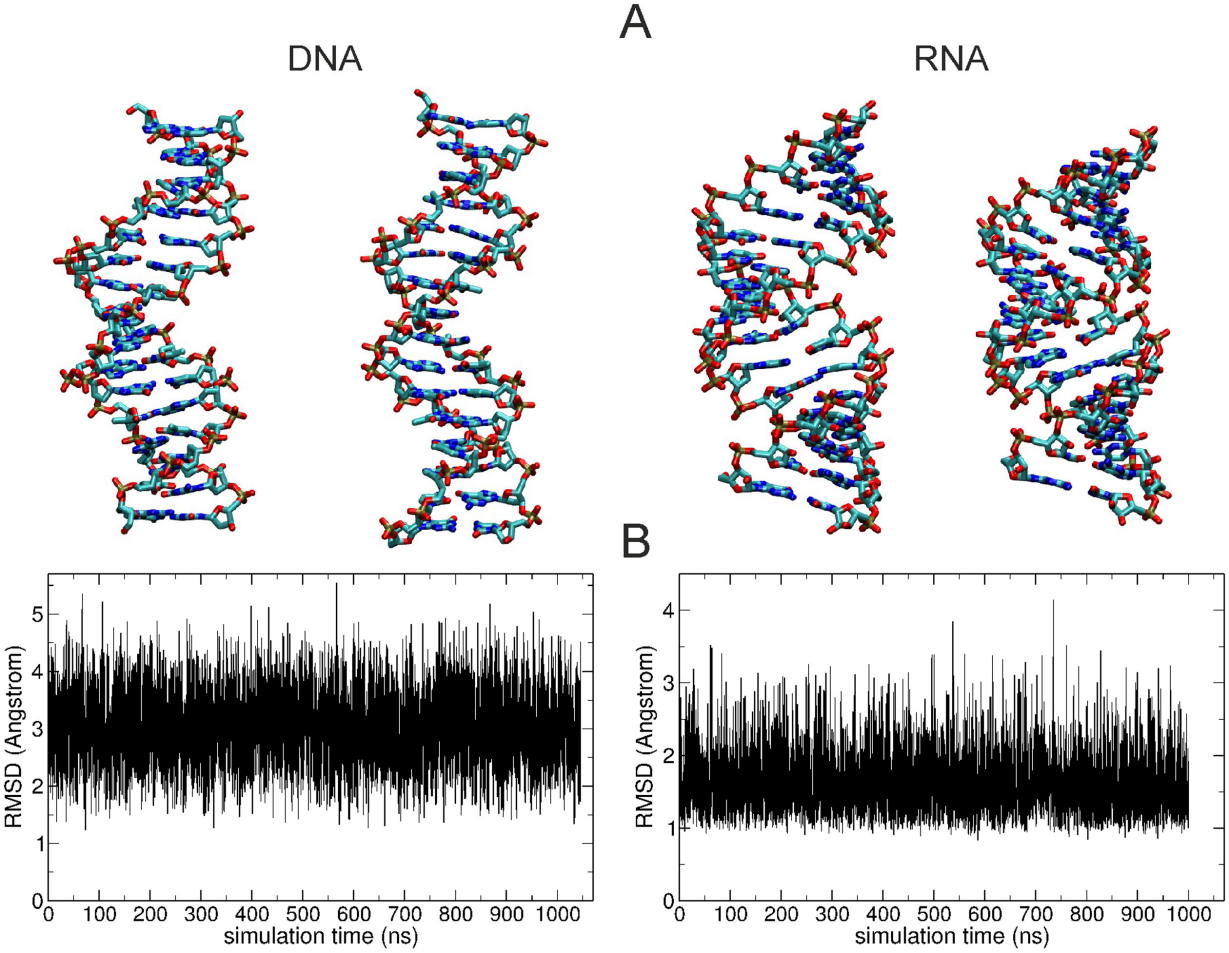
(**A**) Snapshots of undertwisted (left) and overtwisted (right) dsDNA and dsRNA extracted from 1 μs Molecular Dynamics simulations. In case of DNA the overtwisted snapshot indicates a greater extension in the helical (z) axis direction and a smaller diameter compared to the unwound snapshot. For RNA the overtwisted snapshot shows a reduced extension in the z-direction, a more closed major groove and also a reduced diameter compared to an unwound snapshot. (**B**) Root mean square deviation (RMSD) of all heavy atoms of the central 10 base pairs with respect to standard B-form DNA (left plot) and standard A-form RNA (right plot) sampled during the entire data gathering simulations.

**Figure 2.**
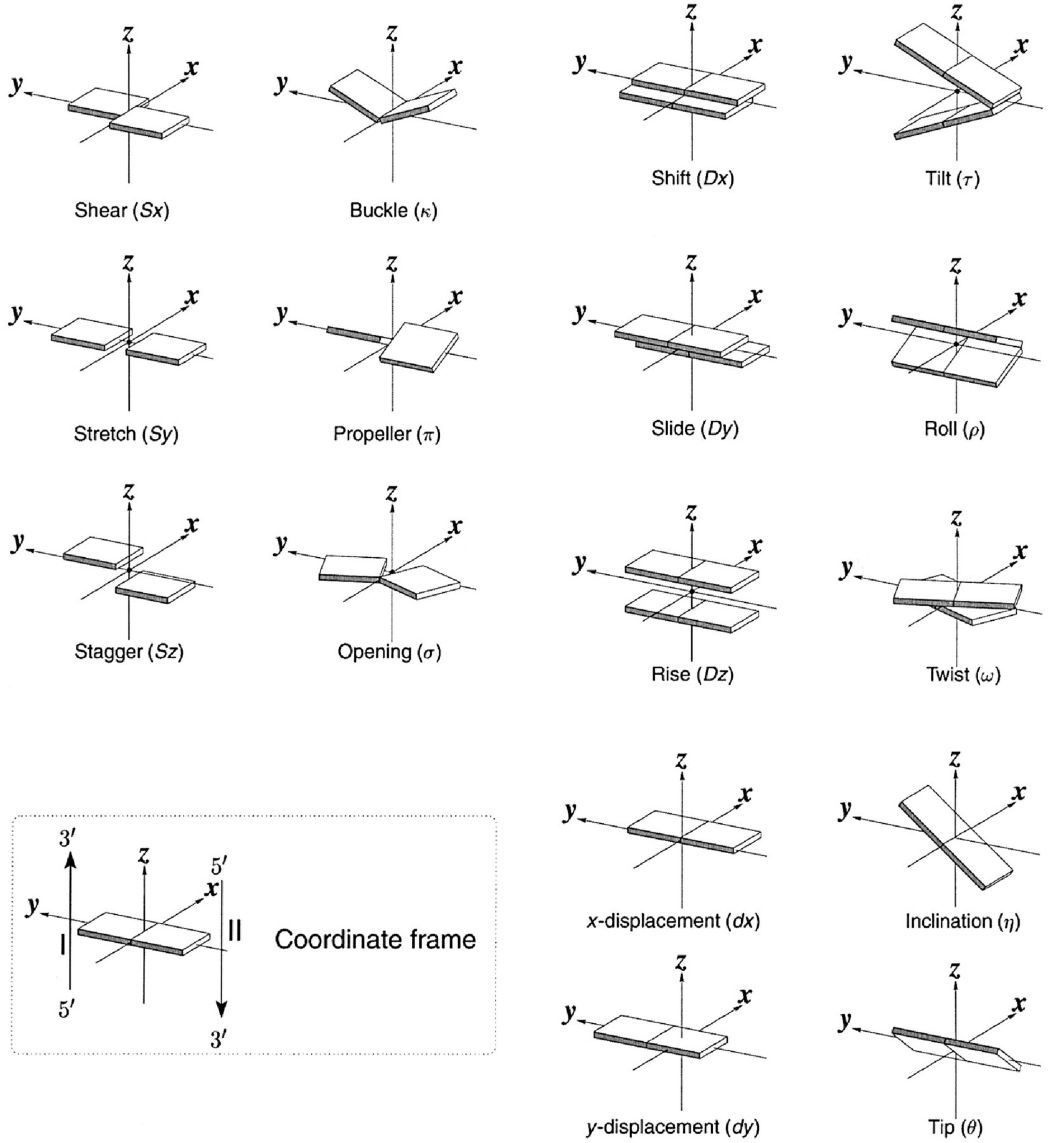
Definition of local base pair parameters (left panels), local base pair step parameters (right panels) and helical base pair axis parameters (lower right panels, in this description helical twist and helical rise correspond to a rotation and translation along a helical axis, respectively) and coordinate frame. Note, that both the set of local base pair step parameters as well as the helical base pair axis parameters offer a complete description of the base pair placements in space (60).

The 1 μs MD simulations of DNA and RNA duplex molecules resulted in sampled conformations that stayed overall close to the B-form or A-from starting structures, respectively (Figure 1). Slightly larger root mean square deviation (RMSD) of the 10 central base pairs with respect to the start structures was observed for dsDNA compared to dsRNA (Figure 1). During the simulations a range of conformations with varying overall twist and extension in the direction of the helical axis were sampled (illustrated by snapshots observed during the simulations, Figure 1). To avoid any influence of the sterically less restricted terminal base pairs the analysis of helical parameters was limited to the 10 central base pairs. Note, that the molecules contain each sequence of possible base pair steps at least once to approximately mimic a random sequence. Neglecting details of the nucleic acid backbone and assuming near rigid geometry of the nucleo-bases allows the description of the placement of each base pair in a duplex structure along a central helical axis by the helical parameters x-displacement (x-disp), y-displacement (y-disp), helical rise, inclination, tip and helical twist (Figure 2). Alternatively, it is also possible to describe a helical duplex structure with respect to the local base pair step parameters shift, slide, rise, tilt, roll and twist (with respect to a local base pair step) (59,60) (Figure 2). The description is completed by six additional intra-base pair helical parameters that describe the internal geometry of bases within each base pair (59,60). We focus on the base pair axis parameters in particular on the relation of helical rise and helical twist since these are the variables that are manipulated and measured in single molecule experiments that twist and stretch duplex molecules (27). The mean helical twist of the central base pair steps extracted from the simulations was 32.0° for DNA and 31.5° for RNA. Whereas the value for RNA is close to standard A-form RNA twist (∼32°) for DNA it is slightly smaller than the standard B-DNA twist of 36° (59). However, it is close to what has been found as average twist (32.5°) in a systematic MD comparison of all possible 136 DNA tetra-nucleotides embedded in duplex molecules using the same force field (49). The observed fluctuations in helical twist (1.1° for DNA and 0.9° for RNA) and rise (0.11 Å for DNA and 0.15 Å for RNA) are compatible with available experimental data on the twist and stretch modulus of DNA and RNA, respectively.

**Figure 3.**
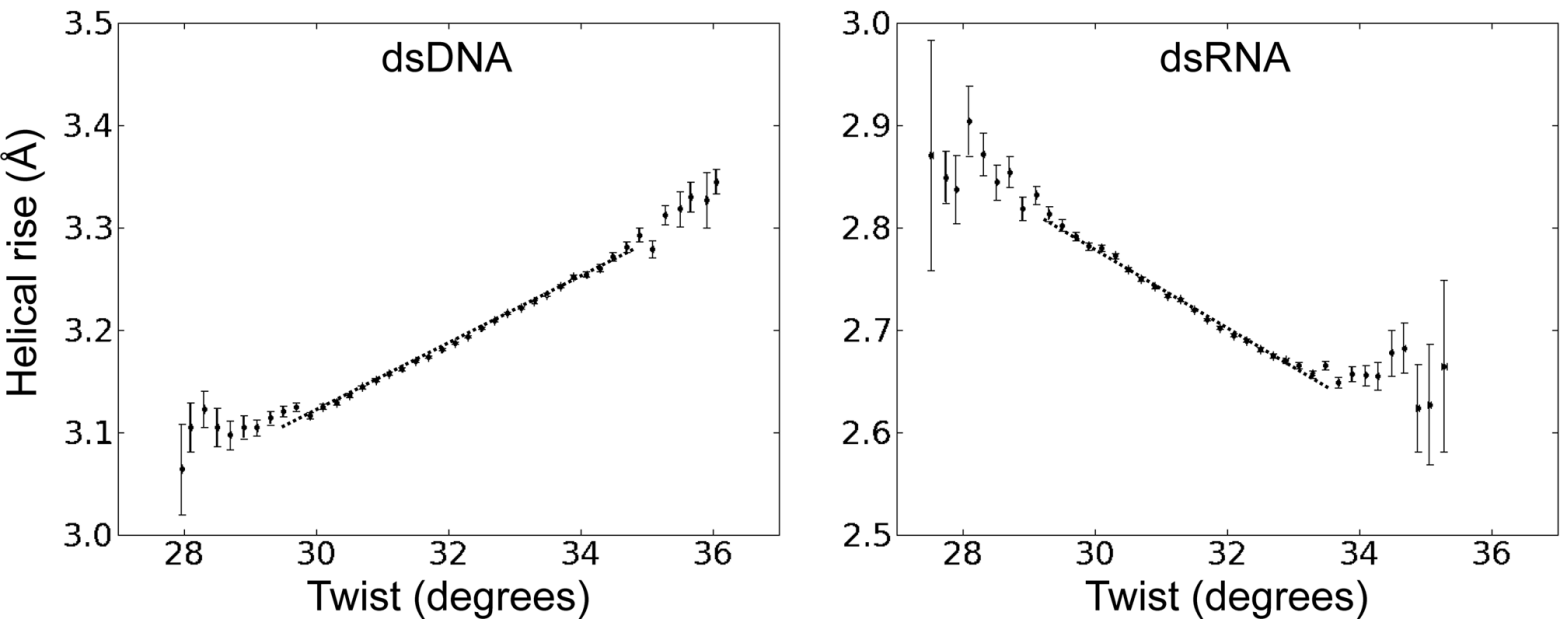
Coupling of helical rise and helical twist variation in unrestraint simulations of dsDNA and dsRNA. The plots were generated from a total of ∼100 000 regularly spaced snapshots (every 10 ps) taken during ∼1 μs unrestraint MD simulations. Recorded helical twist and rise of the 10 central base pair steps were analyzed as the mean helical rise and helical twist within intervals of 0.2°. Error bars for twist and rise were obtained as standard errors of the mean in each interval. The slope of the correlation was extracted from a linear fit to the data for a range of ±1.5° with respect to the average twist (over the entire range of twist values, indicated as dotted line). The slope was 0.032 Å·deg−1 in case of DNA and −0.037 Å·deg^−1^ in case of RNA.

The conformational fluctuations observed during unrestrained MD simulations of the duplexes include correlated motions of important helical degrees of freedom. In the present study we are especially interested in the twist-stretch coupling of DNA and RNA as recent single molecule experiments indicated the opposite behavior for DNA and RNA (27). The twist-stretch coupling can be directly extracted from a correlation plot of the helical rise and helical twist (Figure 3). On the length scale of the present duplex oligonucleotides (10 base pair steps) effects due to bending are negligible. The extraction of a mean helical rise on short intervals of the sampled twist (dots in Figure 3) indicates a linear stretch/twist correlation over a range of twist fluctuations for both DNA and RNA. This is compatible with a near harmonic elastic response of the duplex molecules (see also paragraph on stiffness analysis, below). The correlation allows the extraction of the twist-stretch coupling constant (slope) which gives a value of 0.032 Ådeg^−1^ for DNA and −0.037 Ådeg^−1^ for RNA. This qualitatively different behavior of an opposite sign was also observed experimentally and the coupling constants are in quite good agreement with recent torque tweezer experiments on larger duplexes yielding 0.014 ± 0.003 Ådeg^−1^ for DNA and −0.024 ± 0.001 Ådeg^−1^ for RNA (27), respectively (see also Supplementary Information Table S1).

In the current study the mean twist and rise (and other helical variable) fluctuations were calculated as averages over the 10 central base pairs of the duplexes. It should be emphasized that the contributions from each step are likely sequence dependent (34) and possibly non-additive. For example, the sum of the mean twist fluctuations of individual base pair steps along a segment are not equal to the mean twist fluctuation of a given segment because twist fluctuations are significantly anti-correlated with fluctuations of neighboring base pair steps (local twist fluctuations are larger than the mean twist fluctuation taken over a longer segment) (e.g. 62). Such correlations fall off rapidly beyond nearest neighbors. Since our coordinates are the sums of helical twists and rises (or equivalently, their mean values per step), the correlations between different steps are automatically taken into account.

**Figure 4.**
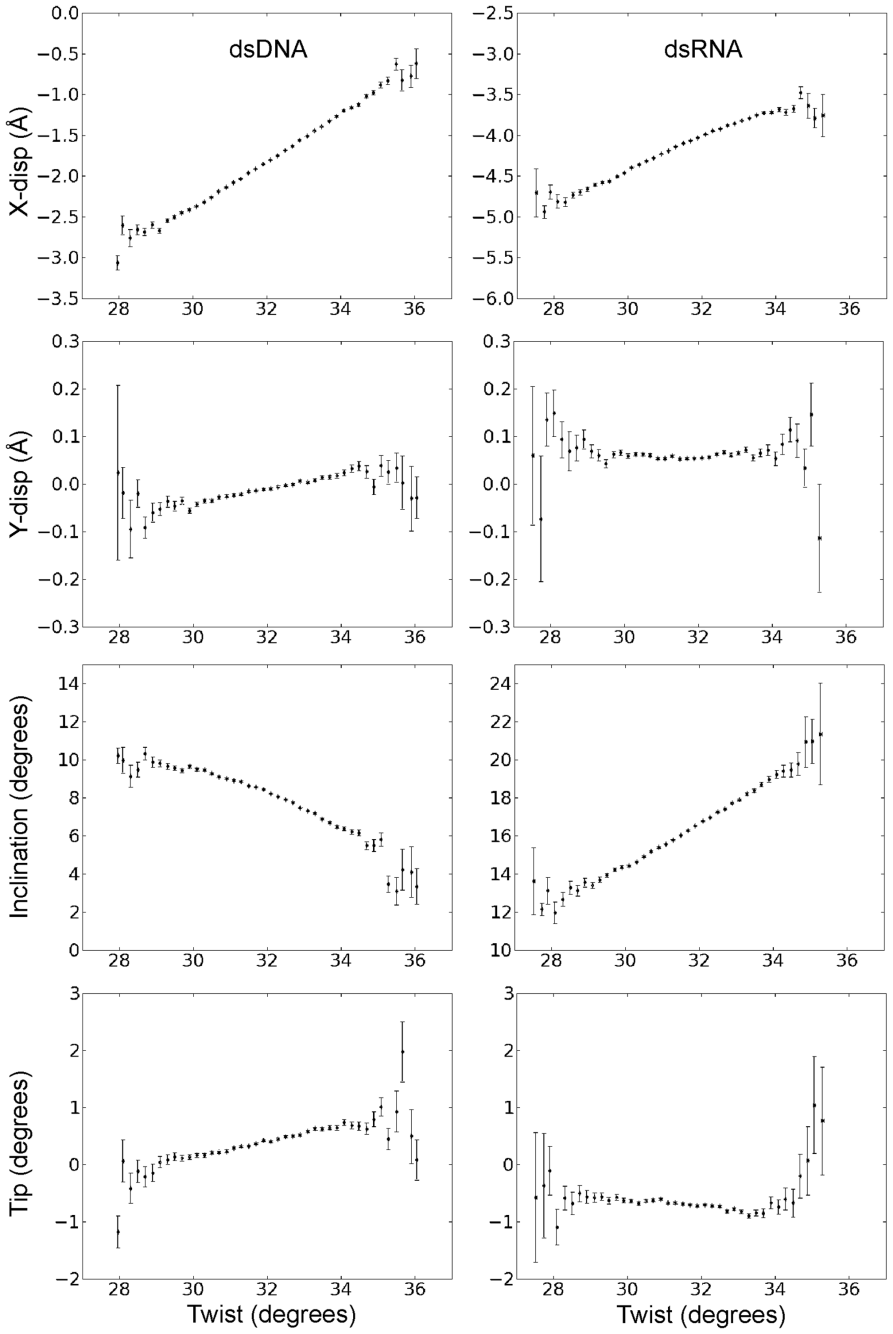
Correlation of helical base pair axis parameters and helical twist during unrestrained MD simulations. The plots were generated in the same way as described in the legend of Figure 3.

**Figure 5.**
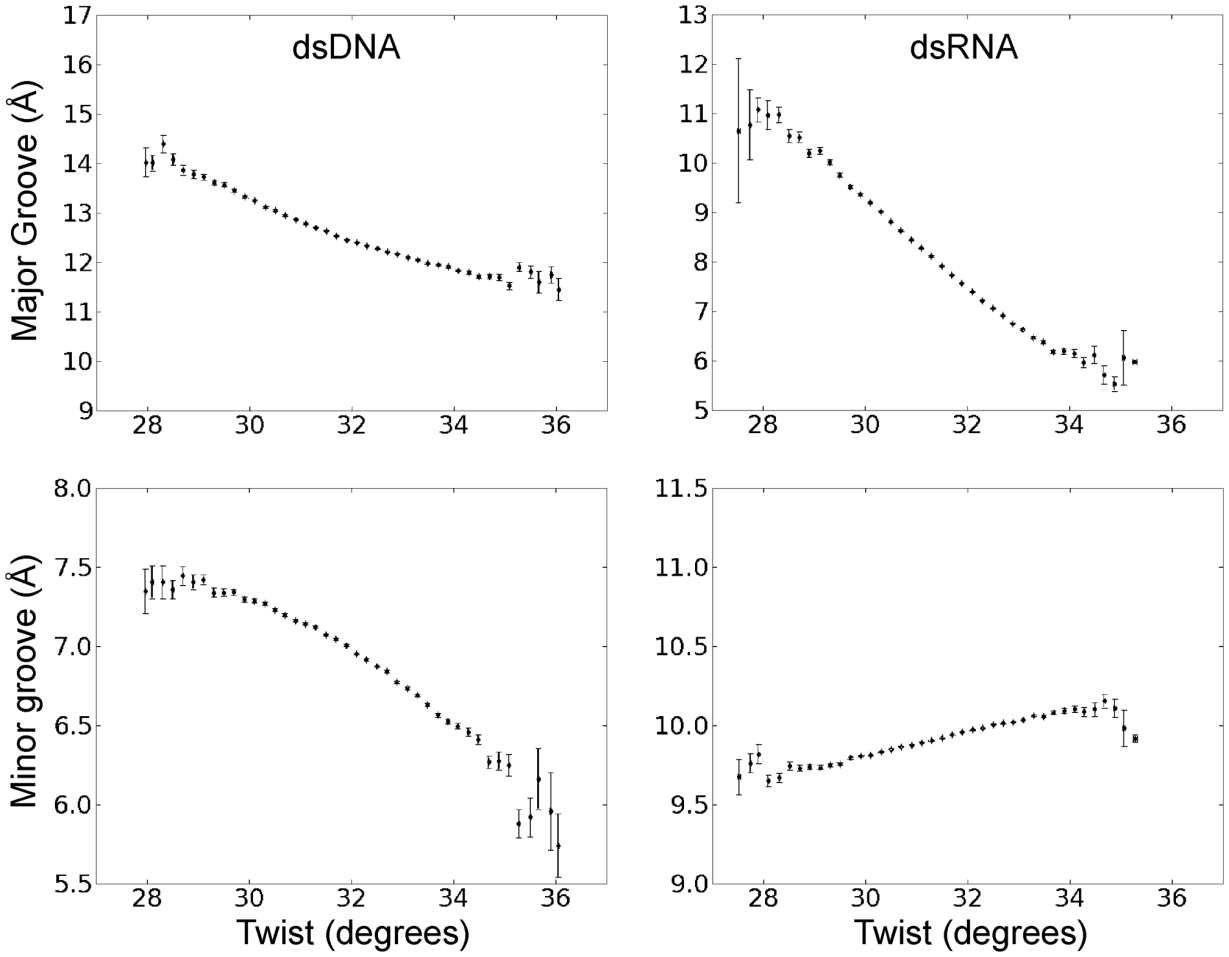
Correlation of major and minor groove width with helical twist during unrestraint MD simulations.

In order to investigate the origin of the different twist-stretch couplings in DNA and RNA, we examine the correlation of other helical variables with respect to helical twist. For the base pair axis parameters no or only very modest correlations of y-disp and Tip with respect to changes in twist were observed for both dsRNA and dsDNA (Figure 4). However, in both cases significant coupled changes of x-disp and base pair inclination with respect to the helical axis were found (Figure 4). The x-disp is negative at low twist angles and the positive x-disp-twist correlation found for both RNA and DNA moves the base pairs on average close to the helical axis upon overwinding (increased twist). Hence, the width of the duplexes shrinks with increasing twist and the effect is more drastic for DNA than RNA (Figure 4). A striking difference between the two duplexes is the coupling of inclination and twist, which is of opposite sign for RNA (positive) and DNA (negative) (Figure 4). The increased inclination of RNA base pairs with increasing twist results in a reduced projection of the distance vector between two neighboring base pairs (proportional to local rise) onto the helical (z-) axis and consequently reduces the extension of the duplex along the helical axis (see also interpretation in the next paragraphs). It also results in a closing of the major groove (Figure 5), which for RNA is sterically more easily possible compared to the minor groove (the minor groove width is nearly independent of twist, Figure 5) making the RNA more compact in all directions.

For DNA the decrease in inclination results in an increase of the projection of the stacking distance between neighboring base pairs on the helical axis and therefore in an increase of the extension along the helical axis. The decrease of inclination causes also a significant reduction of the minor groove width with increasing twist (in contrast to RNA, Figure 5). The observed decrease of the major groove with increasing twist (Figure 5) is counter intuitive and due to the definition of the major groove width (59). If one measures the major groove width just along the helical axis a small increase with twist is observed (not shown).

**Figure 6.**
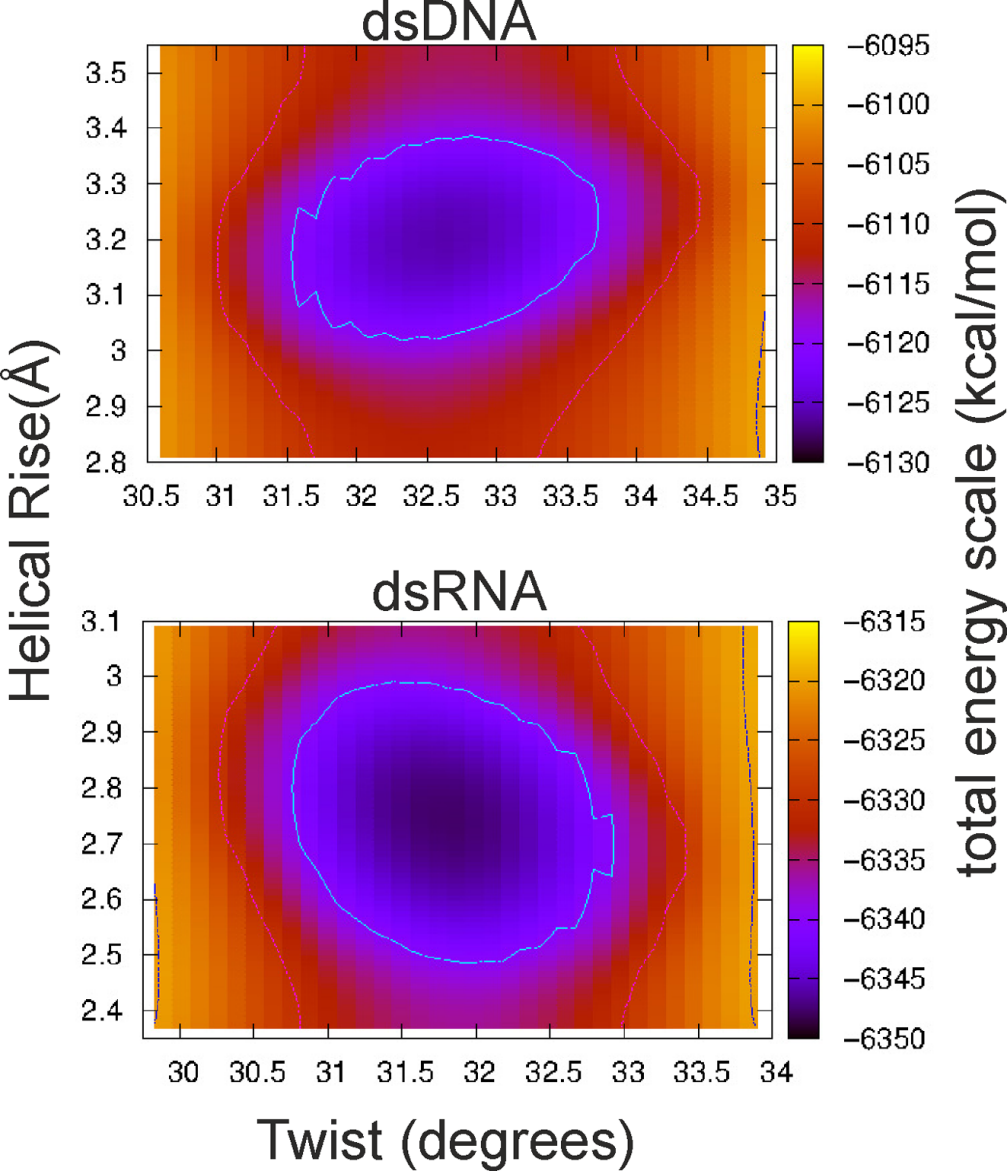
Histogram of the calculated total energy (using an implicit solvent model, see Methods) of sampled dsDNA and dsRNA conformations (in unrestraint simulations) versus mean helical twist and mean helical rise. The color-coded total energy is shown for the range of ∼35 kcal·mol^-1^ with respect to the lowest energy (same color for larger energies).

In order to check if specific energetic contributions are responsible for the observed opposite twist-stretch coupling of RNA and DNA we analyzed the mean energies of the sampled states using the MM/GBSA method. For better convergence of mean energies the solvent is treated as a continuum in this approach and the extracted total energies can only be considered as rough estimates. Nevertheless, the calculated energy landscape versus twist and rise qualitatively reflects the twist-stretch coupling extracted directly from the simulations (Figure 6). The calculations also clearly show that specific combinations of helical twist and helical rise are energetically unfavorable: in case of DNA a regime of low twist (∼30°) and high rise (>3.4 Å) as well as high twist (>34°) and low rise (<3.2 Å) are energetically strongly disfavored and the opposite is true for RNA (Figure 6). Inspection of energetic contributions (Supplementary Figure S1) indicates that bonded terms show only little variation with twist. Similarly, the average hydrogen bonding interactions (Supplementary Figure S2) and average local rise (i.e. stacking distance within a base pair step) are not coupled to twist. This result shows that the observed twist-stretch deformation is possible without any drastic changes in sterical strain. Also, all other contributions show similar trends for RNA and DNA with Lennard–Jones interactions becoming more favorable and Coulombic contributions opposing an increase in twist (Supplementary Figure S1). These latter two observations, however, clearly demonstrate that both DNA and RNA become more compact with increasing twist (e.g. resulting in stronger phosphate group repulsions and overall more attractive dispersion interactions). However, no single energy term can be related to the opposite RNA/DNA twist-stretch couplings and it appears to be determined by a balance of several contributions.

In summary, the analysis of the unrestrained MD simulations semi-quantitatively reproduces the experimentally observed opposite twist-stretch coupling of RNA and DNA. Overwinding results in both cases in a reduction of the magnitude of the x-disp variable (reduction of the duplex radius). However, only for DNA does it result in an increase of the extension along the helical axis coupled to more positive values of base pair inclination. In the case of RNA, an increase in twist results in a strong reduction of the major groove size (reducing the ‘empty’ space between backbone strands flanking the major groove) and a reduction of the extension along the helical axis.

Other significant changes in helical coordinates coupled to changes in helical twist concern changes in roll and propeller twist (see Supplementary Information Figures S3–S5). These changes also modulate the global response to twist stress but show mostly the same sign and approximately the same magnitude for dsRNA and dsDNA.

### Analysis of DNA stiffness

The twist-stretch coupling was also investigated using a harmonic stiffness model parameterized from unrestrained MD simulations, as described in the Methods. The coupling coefficient for the duplex elongation upon imposed twisting (Equation 8), using the 3DNA helical twist and helical rise, was found to be 0.032 Ådeg^−1^ for DNA and -0.033 Ådeg^−1^ for RNA, very close to the values found by linear regression and in reasonable agreement with experiment (see above). If the Curves+ helical coordinates were used instead (as used in the linear regression analysis), very similar values of 0.031 Ådeg^−1^ for DNA and -0.036 Ådeg^−1^ for RNA were obtained. The complementary coupling describing the change of twist upon imposed elongation (Equation 9) was (3DNA/Curves+) 2.38/3.57 degÅ^−1^ for DNA and −1.53/−1.54 degÅ^−1^ for RNA. Thus, DNA overwinds when stretched (15), while RNA underwinds (27). The correct sign of the coupling coefficients and the nearly quantitative agreement with experiment demonstrates the capability of unrestrained MD simulations, and of the harmonic model parameterized from them, to capture detailed aspects of nucleic acids mechanics.

### Molecular dynamics simulations including a torque restraining the total twist

**Figure 7.**
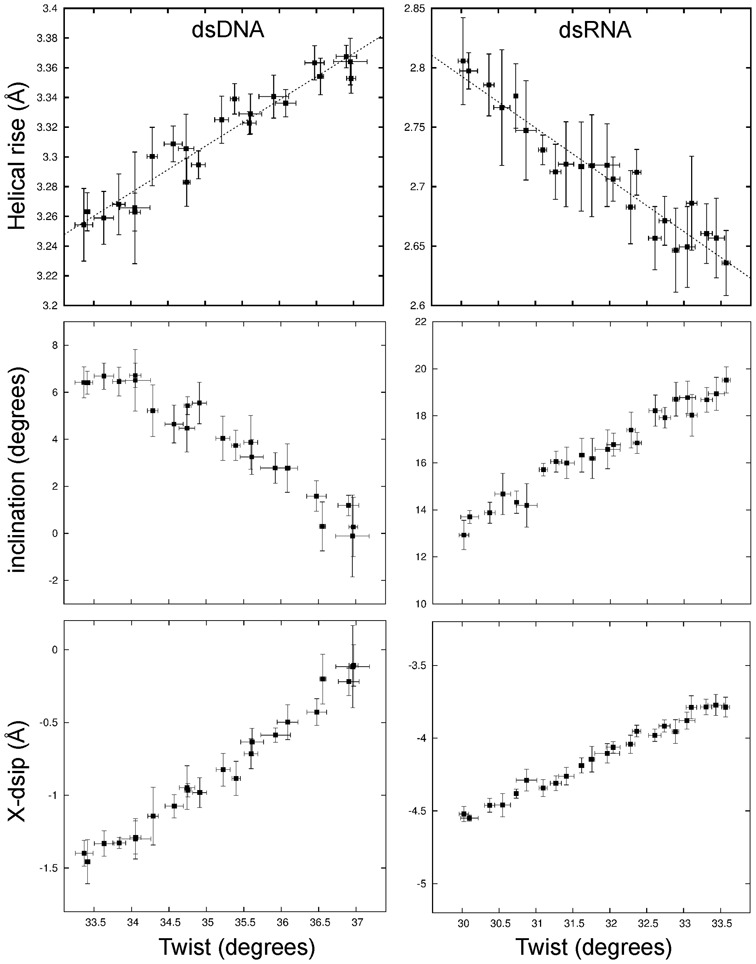
Variation of helical rise, inclination and x-disp during simulations with a torque restraint on the total twist of the DNA (left panels) and RNA (right panels). The mean twist per base pair step was changed in step of ∼0.15° employing a soft harmonic potential on the total twist (see Methods for details) and the resulting mean rise, inclination and x-disp were recorded. Errors were estimated by splitting the data (10 ns simulation time per twist step) into five intervals and separate calculation of mean helical parameters and the standard deviation over these intervals.

Instead of extracting variances and covariances of conformational fluctuations directly from unrestrained MD simulations (see previous sections) it is also possible to use external forces and torques for deforming a duplex along a selected direction. In order to mimic more closely the experimental setup to investigate twist deformations in dsDNA and dsRNA a quadratic restraining potential for controlling the overall twist was added to the force field. The dihedral angle penalty potential was defined by the (desoxy)ribose C1’ atoms of the 3rd and 14th base pairs (four atoms) and encompassed the central 12 base pair steps (only the 10 central steps were used for analysis). The adjustment of the reference angle in the quadratic potential allowed controlling the total twist of the central segment and can be translated into a mean twist change per base pair step (extracted by Curves+). A similar setup was used in a previous study to systematically investigate the sequence dependence of twist flexibility of DNA (58). For each applied external total restraint twist the average twist per base pair was evaluated using Curves+ (59). The twist restraining simulations were focused on a range of mean twist angles of ∼33° to 37° for DNA and ∼30° to 33.5° for RNA which encompassed the observed equilibrium twist in the unrestrained MD simulations but also the twist found in standard A- and B-form duplexes. Similar to an experimental torque tweezer experiment this approach allows us to adjust the total helical twist to a desired narrow range and to extract the corresponding equilibrium helical rise (and other helical parameters). A linear relation between helical rise and twist was obtained (Figure 7). The slope of the calculated twist-stretch correlation was positive for dsDNA (0.029 Ådeg^−1^) and negative in case of dsRNA (−0.040 Ådeg^−1^) and in near quantitative agreement with the correlations obtained from unrestrained simulations on the same duplexes (see above). Also, the relation of other helical parameters like inclination or x-disp with respect to the restraint helical twist resulted in near quantitative agreement with the correlations obtained from the unrestrained MD simulations (see previous sections) supporting the robustness of the present results. Correlations of other helical variables with twist are given in the Supplementary Information section (Supplementary Figures S5 and S7).

### Molecular mechanism of coupled twist-stretch deformations

The most striking changes of helical variables coupled to twist are the x-disp and the change of base pair inclination relative to the helical axis. Whereas the change in x-disp is similar in sign for dsDNA and dsRNA (with smaller magnitude) the change in inclination is of opposite sign (upon twist change). In case of A-form RNA the helix is actually built like a spiral stair with the helical axis in the middle not touching the base pairs (stairs) and the stairs already positively inclined relative to the helical axis. For dsRNA the positive inclination becomes even more positive upon overwinding of the helix. Coupled to a change in x-disp this actually brings the backbone of the two strands closer together upon overwinding by (strongly) decreasing the major groove width.

Using the program Jumna (61) it is possible to generate an RNA or DNA duplex using the base pair helical axis parameters as input. In addition, it is possible to sterically relax the generated structure in all backbone and helical variables but lock (keep) selected helical variables constant. In this way it is possible to analyze qualitatively the interplay of the helical variables twist, rise, inclination and x-disp.

**Figure 8.**
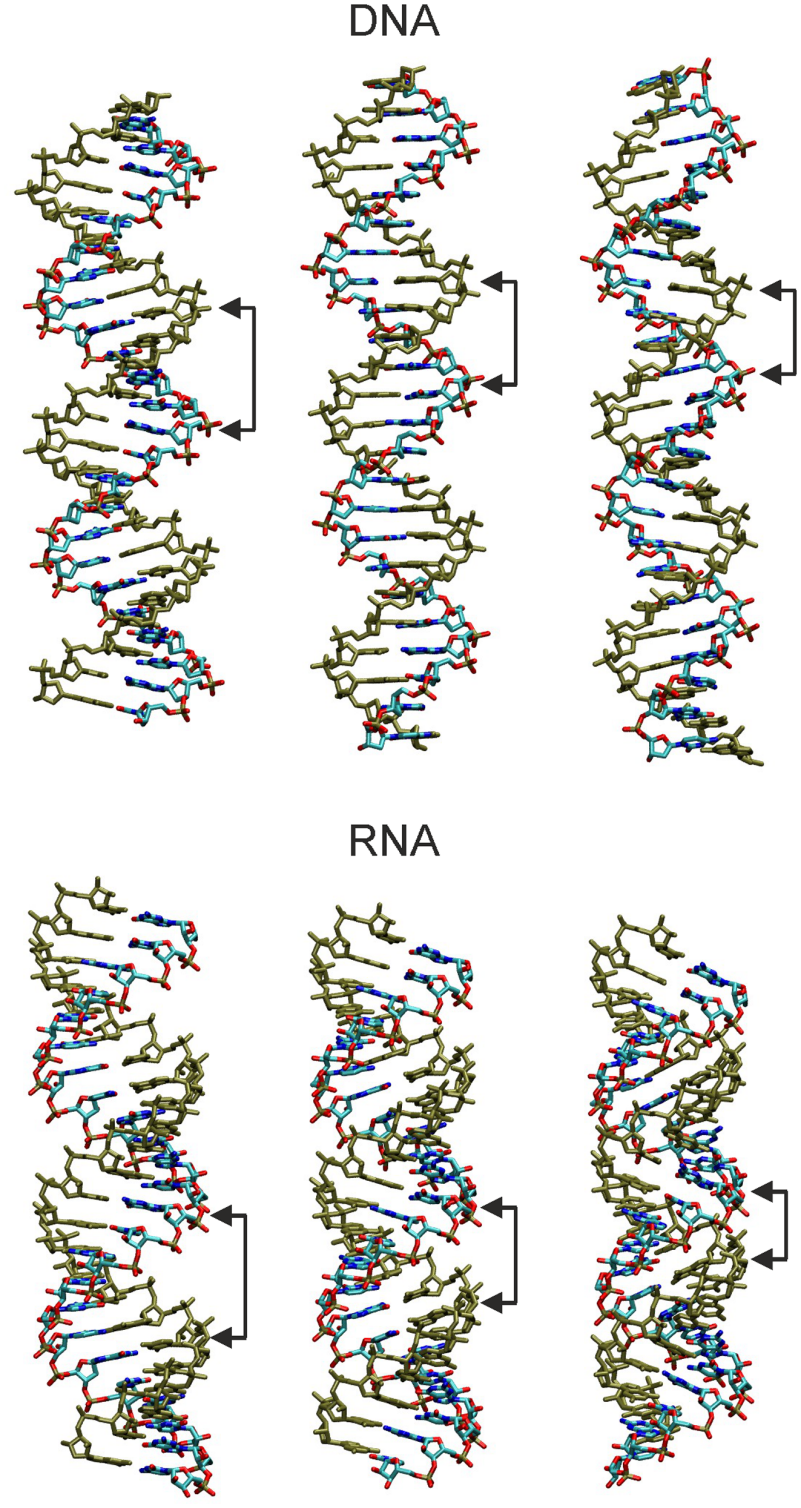
Illustration of twist-stretch coupling in DNA and RNA induced by locking base pair inclination and x-disp. Energy minimized structures were generated in helical and nucleic acid backbone coordinates using Jumna (61) keeping x-disp and inclination locked (see Table 1). Structures in the middle correspond to relaxed/minimized canonical B-form (DNA) and A-form (RNA) structures (keeping x-disp and inclination locked to canonical values). (left panels) DNA locked to negative x-disp and positive inclination (producing underwound and shortened duplex, see Table 1); RNA locked to more negative x-disp and reduced (positive) inclination (resulting in unwinding and a more extended helix with increased major groove).(right panels) DNA locked to positive x-disp and negative inclination (yielding reduced minor groove width, increased helical rise and increased twist; opposite effect seen for RNA (less negative x-disp and increased inclination produces increased twist and shrinking of the helical extension).

In case of RNA canonical starting values for twist (32.5°) and rise (2.6 Å) were used combined with an x-disp = −4.4 Å and incl = 17.5° close to the mean values seen in the MD simulations. In addition, two deformed start states with more negative x-disp = −4.8 Å and reduced incl = 10.5° or x-disp = −4.0 Å and incl = 25.5°, respectively, were energy minimized keeping x-disp and inclination locked. Indeed, the sterically optimized RNA locked to a more negative x-disp and reduced inclination resulted in an unwinding of the helix and increased helical rise (Table 1) and an open major groove (Figure 8). The opposite was observed for the relaxation of the other deformed start structure compared to the (relaxed) reference structure generating an overwound (overtwisted) RNA molecules with also strongly reduced helical rise and reduced major groove (very similar to the results of the MD simulations, Figure 8). Hence, the twist-stretch coupling observed for RNA in MD simulations can also be achieved by using x-disp and incl as input variables (allowing twist and rise to adopt the sterically most favorable combination) demonstrating the interplay between the helical variables. It is important to note that the same energy minimization results (to within an RMSD of < 0.3 Å) were achieved for A-DNA versus A-RNA. This provides strong evidence that the helical topology, rather than the presence of an additional hydroxyl group or the uridine instead of adenine base in RNA, mediates the opposite twist-stretch coupling of B-DNA and A-RNA.

**Table 1.** Energy minimization of dsDNA and dsRNA model structures with sequence (GC)_12_ using Jumna (61) qu

DNA	Structure^a^	<x-disp(Å)>	<y-disp(Å)>	<hrise(Å)>	<Incl.(°)>	<Tip(°)>	<htwist(°)>
Ref.	start/final	**0.0/0.01**	0.0/0.0	3.4/3.31	**0.0/0.01**	0.0/0.0	36.0/40.1
deform1	start/final	**−1.4/−1.4**	0.0/0.0	3.4/3.2	**10.0/10.0**	0.0/0.0	36.0/38.3
deform2	start/final	**1.4/1.4**	0.0/0.0	3.4/3.37	**−10.0/−10.0**	0.0/0.0	36.0/42.7
RNA	structure						
Ref.	start/final	**−4.4/−4.4**	0.0/0.0	2.6/2.67	**17.5/0.0**	0.0/0.0	32.7/34.7
deform1	start/final	**−4.8/−4.8**	0.0/0.0	2.6/2.89	**10.5/10.5**	0.0/0.0	32.7/32.2
deform2	start/final	**−4.0/−4.0**	0.0/0.0	2.6/2.51	**25.5/25.5**	0.0/0.0	32.7/38.1

^a^start indicates the helical parameters (assigned to each base as input); final indicates the average after energy minimization.

As discussed in the Introduction section, overtwisting of a duplex (e.g. by applying a torque) tends to bring the two strands closer together. For RNA the sterically most favorably way to bring the backbone strands closer together upon overwinding is a closing of the major groove (achieved by adjusting inclination and x-disp).

In case of dsDNA the helical axis runs approximately through the middle of the base pairs. The sugar phosphate backbone of the two strands comes here closer together along the deeper and more flexible minor groove (compared to RNA). A torque acting on the ends of a helical segment on both strands tends to decrease the width of the minor groove to reduce the distance between backbones on the two strands. A closing of the minor groove can be achieved by changing the inclination of each base pair in the negative direction (compared to RNA) and appropriate adjustment of the x-disp. In order to mimic this process one can again employ the Jumna program. In this case a standard B-DNA (locked zero inclination and locked zero x-disp) served as reference. A start structure with negative x-disp (locked to −1.4 Å) and positive inclination (locked to 10°) resulted in an energy optimized structure with reduced helical rise and reduced twist (Table 1) and a more open minor groove compared to the reference (Figure 6). The opposite behavior is observed for the choice of a negative inclination (−10°) and positive x-disp (1.4 Å) resulting in a more extended helix (increased helical rise), increased twist and reduced minor groove (Table 1, Figure 8).

**Figure 9.**
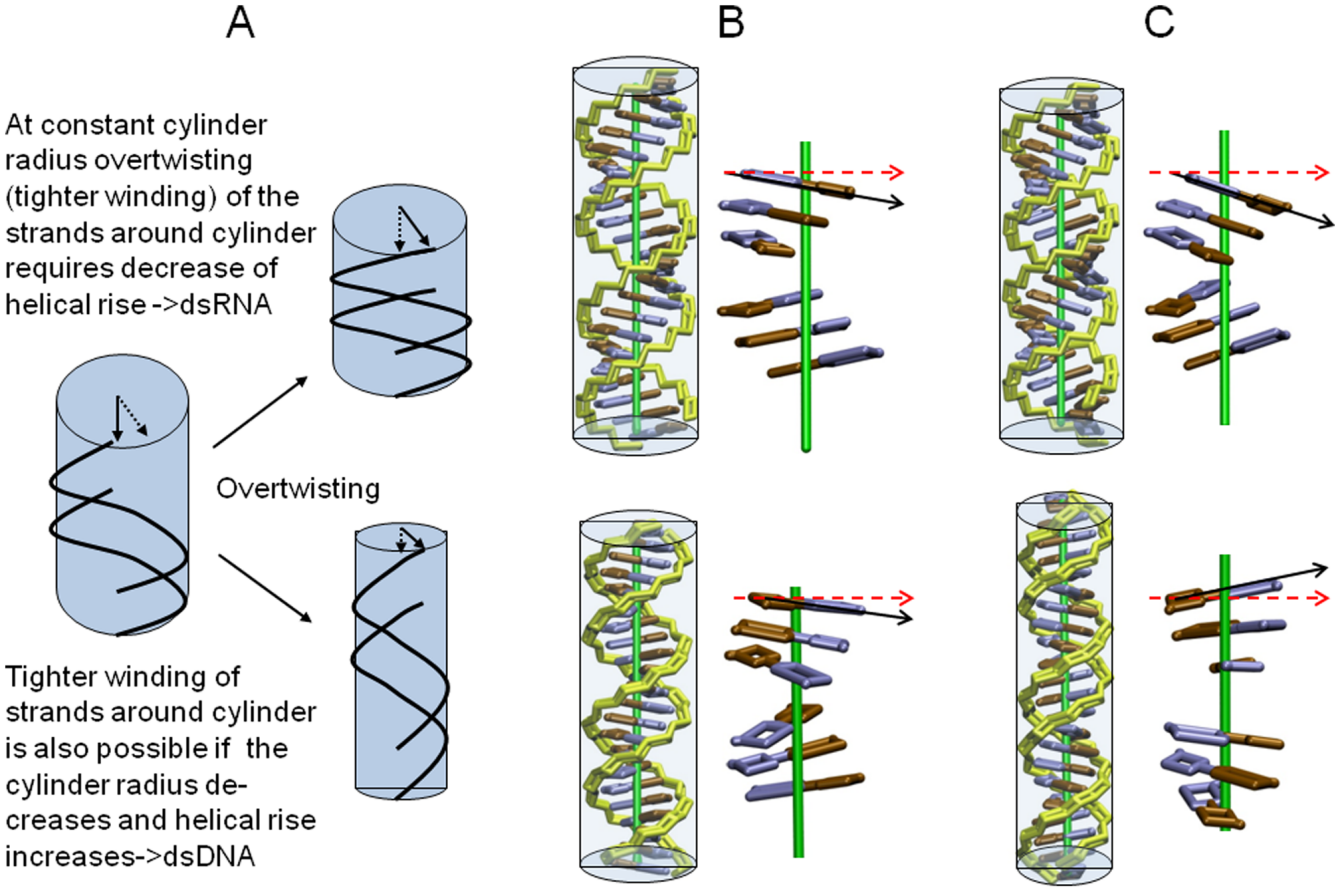
(**A**) Effect of tighter wrapping (overtwisting) of two simplified nucleic acid strands (black lines) around a cylinder (in light blue) at constant radius and with decreasing cylinder radius. The simplified RNA and DNA representations have been generated based on the helical parameters sampled at low twist values (∼28°, **B**) and high twist values (∼34°, **C**). Backbone is shown in yellow and bases of one strand in blue and brown, respectively. Enlarged blocks of three base pairs separated by approximately half a helical turn are shown without the backbone for clarity. In case of RNA (upper panels in **B**, **C**) the diameter of the double helix decreases only slightly and the length of the helix decreases with increasing twist. This is achieved with a minimum of sterical strain by a more acute Inclination (compare black and red arrows in **B**, **C**) and a slide motion (without disturbing the base pair geometry) resulting in a more narrow minor groove and compaction along the helical axis (black arrows in **C**). For DNA the increase of twist results in a significant narrowing of the helix (reflected by the large change in x-disp). With a minimum of sterical strain this is achieved by a change of inclination in the opposite direction compared to RNA (compare black and red reference arrows in **B**, **C**) which extends the helix (black arrows in **C**).

Finally, a model for the opposite twist-stretch coupling in DNA and RNA can be extracted from our simulation results (illustrated in Figure 9). In order to increase the winding (helical twist) of two helical strands around a cylinder of constant radius the helical rise needs to decrease unless we stretch the nucleic acid backbone (resulting in a large strain energy). This case largely reflects the behavior of RNA (although the diameter of RNA slightly shrinks with increasing twist due to the change in x-disp). The shrinking of the length of the cylinder that embeds RNA with increasing twist is sterically easily possible because the large major groove provides sufficient space to accommodate the RNA atoms. The mechanism to close the major groove and to reduce the helical rise is due to an inclination motion (toward more negative inclination, as illustrated in Figure 9). This is for the A-form RNA topology the sterically best compromise (does not create any significant sterical overlap or strain). A second possible response to allow for overwinding of the strands around a cylinder is to reduce the radius of the cylinder. However, this strongly reduces the available space for the nucleic acid (smaller radius reduces cylinder volume). Consequently, in this case the molecule needs to stretch out and to fill a longer cylinder. Exactly this behavior was observed in the simulations of DNA (illustrated in Figure 9). It is sterically possible by a change of incl in the opposite direction as observed for RNA, resulting in a closing of the minor groove and helix extension.

## CONCLUSIONS

DsDNA and dsRNA exhibit only modest quantitative differences in their global bending, stretching and twisting flexibilities. However, in terms of the coupling of twist and stretch the opposite behavior has been found for dsDNA and dsRNA (27). This is a striking difference which could be of importance for understanding packing of DNA under the influence of torsional stress and for long-range stretching deformation of helical RNA molecules in large protein–RNA assemblies (e.g. ribosome) triggered by a twist deformation of RNA. The twist–stretch coupling has potentially important biological implications, such as for how mutations affect binding sites, because a base pair deletion or insertion changes not only the length but also the twist of the target sequence, changes that need to be compensated by distortions of the nucleic acid upon protein binding. In the present study the opposite twist-stretch coupling for dsDNA and dsRNA (of the corresponding sequence) was replicated in extensive unrestrained explicit solvent MD simulations and in simulations with an external torque applied to the terminal base pairs of the duplexes. The extracted twist-stretch coupling constants were found to be in quite good agreement with available experimental data indicating that current molecular mechanics force fields and harmonic models parametrized from MD are useful to characterize even such fine details of the global flexibility of double stranded nucleic acids and allow a clear distinction of the dynamic behavior of DNA versus RNA. Analysis of the change of helical parameters coupled to the change in twist allowed us to trace the origin of the differential twist-stretching behavior of DNA and RNA. The overwinding of a double helix consisting of two nucleic acid strands enforces a reduction of the distance between the backbones of the two strands. In case of RNA this leads to a reduction of the size of the major groove, reducing the distance between phosphate groups along the major groove (but not along the minor groove). As a consequence the inclination of the base pairs increases (becomes more positive) reducing the effective (projected rise) on the helical axis. For DNA the minor groove width decreases resulting in a change of inclination in the opposite direction and (together with a sliding the base pairs) an increase in the projected rise along the helical axis.

For the current study a ‘mixed’ sequence was employed that included all possible base pair steps along a helix. In the future, different sequences could be studied to investigate the sequence dependence of twist-stretch coupling more comprehensively.

## Supplementary Material

SUPPLEMENTARY DATA
