# Supplementary material for: Explaining the striking difference in twist-stretch coupling between DNA and RNA: A comparative molecular dynamics analysis
Source: Nucleic Acids Res. 2015 Oct 12;43(21):10143–56. doi: 10.1093/nar/gkv1028 (PMC4666353; doi:10.1093/nar/gkv1028)
Supplement: SUPPLEMENTARY DATA [file supp_43_21_10143__index.html]

Explaining the striking difference in twist-stretch coupling between DNA and RNA: A comparative molecular dynamics analysis — SUPPLEMENTARY DATA 

# Explaining the striking difference in twist-stretch coupling between DNA and RNA: A comparative molecular dynamics analysis

## SUPPLEMENTARY DATA

- SUPPLEMENTARY DATA
