# Supplementary material for: Explaining the striking difference in twist-stretch coupling between DNA and RNA: A comparative molecular dynamics analysis
Source: Nucleic Acids Res. 2015 Oct 12;43(21):10143–56. doi: 10.1093/nar/gkv1028 (PMC4666353; doi:10.1093/nar/gkv1028)
Supplement: SUPPLEMENTARY DATA [file supp_gkv1028_nar-02042-f-2015-File011.pdf]

# Supplementary Information

## Explaining the striking difference in twist-stretch coupling between DNA and RNA: A comparative molecular dynamics analysis

Korbinian Liebl[a], Tomáš Dršata[b,c], Filip Lankaš[b,d], Jan Lipfert[e] and Martin Zacharias[a]*

[a]) Physik-Department T38, Technische Universität München, James-Franck-Strasse, D-85748 Garching, Germany

[b]) Institute of Organic Chemistry and Biochemistry, Academy of Sciences of the Czech Republic, Flemingovo nám. 2, 166 10 Prague, Czech Republic

[c]) Department of Physical and Macromolecular Chemistry, Faculty of Science, Charles University Prague, Albertov 6, 128 43 Prague, Czech Republic

[d]) Laboratory of Informatics and Chemistry, University of Chemistry and Technology Prague, Technická 5, 166 28 Prague, Czech Republic

[e]) Department of Physics, Center for Nanoscience (CeNS), and Nanosystems Initiative Munich (NIM), Ludwig-Maximilian-University Munich, 80799 Munich, Germany

**Table S1.** Overview on experimental measurements of the twist-stretch coupling for double-stranded DNA and double-stranded RNA.

| **Measurement** | Twist stretch coupling: change in length per change in twist (Å/deg) | Twist-stretch coupling parameter *D* (unitless) |
|---|---|---|
| Double-stranded DNA; rotor bead assay [1] | 0.014 ± 0.003 | −22 ± 4.9 |
| Double-stranded DNA; magnetic tweezers [2] | 0.012 ± 0.006 | −17 ± 7 |
| Double-stranded DNA; optical torque wrench [3] | N.A. | −21 ± 1 |
| Double-stranded DNA; magnetic tweezers [4] | 0.012 ± 0.003 | −17 ± 5 |
| Double-stranded RNA;magnetic tweezers [4] | −0.024 ± 0.001 | 11.5 ± 3.3 |


1. Gore, J., Bryant, Z., Nollmann, M., Le, M.U., Cozzarelli, N.R. and Bustamante, C. (2006) DNA overwinds when stretched. *Nature*, **442**, 836-839.

2. Lionnet, T., Joubaud, S., Lavery, R., Bensimon, D. and Croquette, V. (2006) Wringing out DNA. *Phys Rev Lett*, **96**, 178102.

3. Sheinin, M.Y. and Wang, M.D. (2009) Twist-stretch coupling and phase transition during DNA supercoiling. *Phys Chem Chem Phys*, **11**, 4800-4803.

4. Lipfert, J., Skinner, G.M., Keegstra, J.M., Hensgens, T., Jager, T., Dulin, D., Kober, M., Yu, Z., Donkers, S.P., Chou, F.C. *et al.* (2014) Double-stranded RNA under force and torque: Similarities to and striking differences from double-stranded DNA. *Proc Natl Acad Sci U S A*, **111**, 15408-15413.

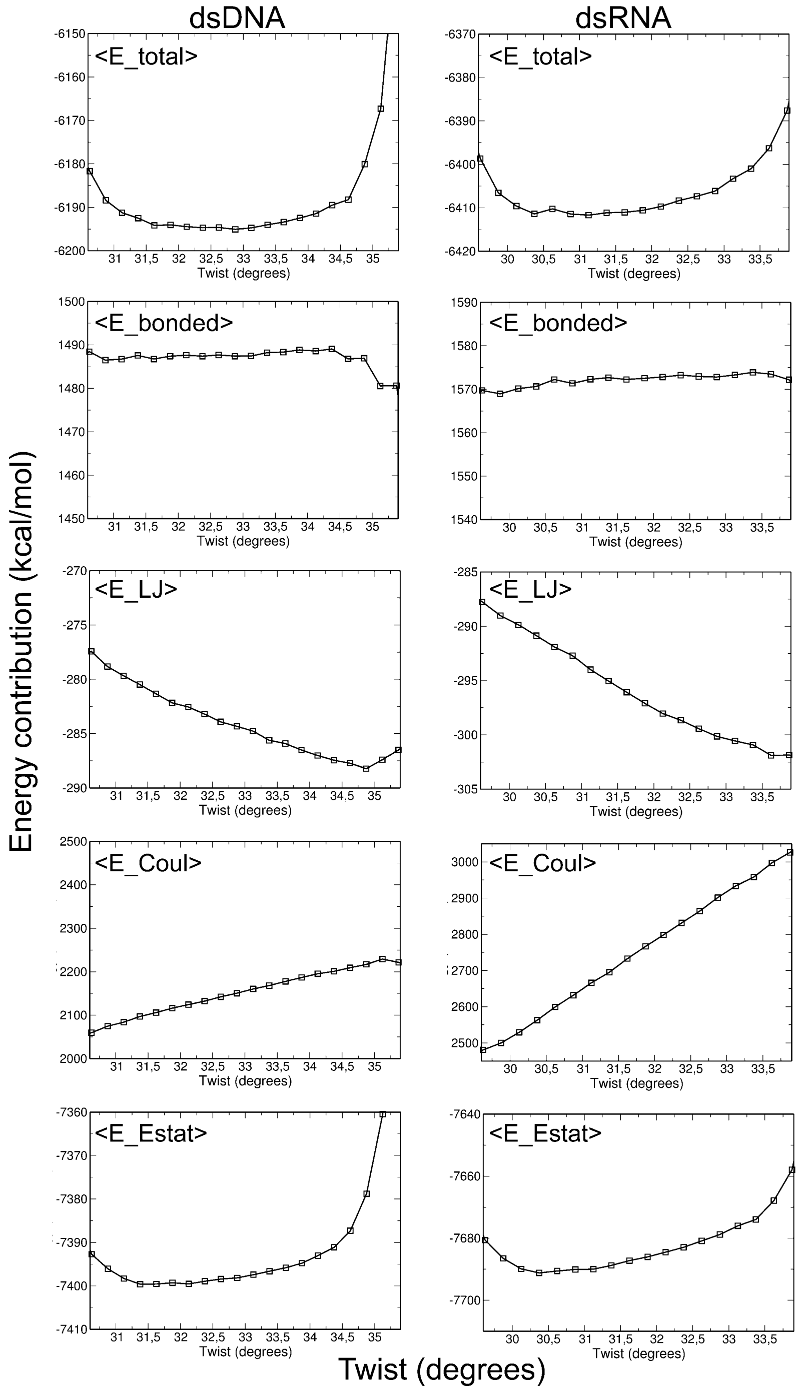


**Figure S1.** Average energy contributions vs. mean twist of sampled states during 1 μs unrestraint simulations of DNA and RNA. Snapshots were evaluated using the MMGBSA approach (see Methods). The mean total energy (<E_total>) is the sum of all bonded contributions (<E_bonded>) such as bond length, bond angle and dihedral contributions and Lennard-Jones (<E_LJ) as well as electrostatic contributions (<E_Estat>). The electrostatic contribution is the sum of Coulomb interactions (<E_Coul>) and the Generalized Born solvation term (not shown, represents the difference between E_Estat and E_Coul).

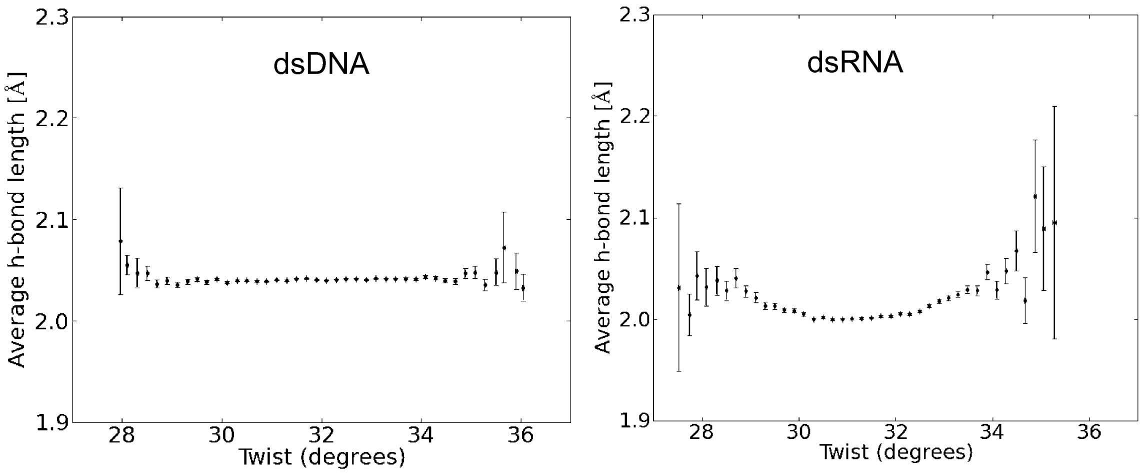


**Figure S2.** Correlation of the mean hydrogen bond length of Watson Crick base pairs vs. twist during unrestraint MD-simulations.

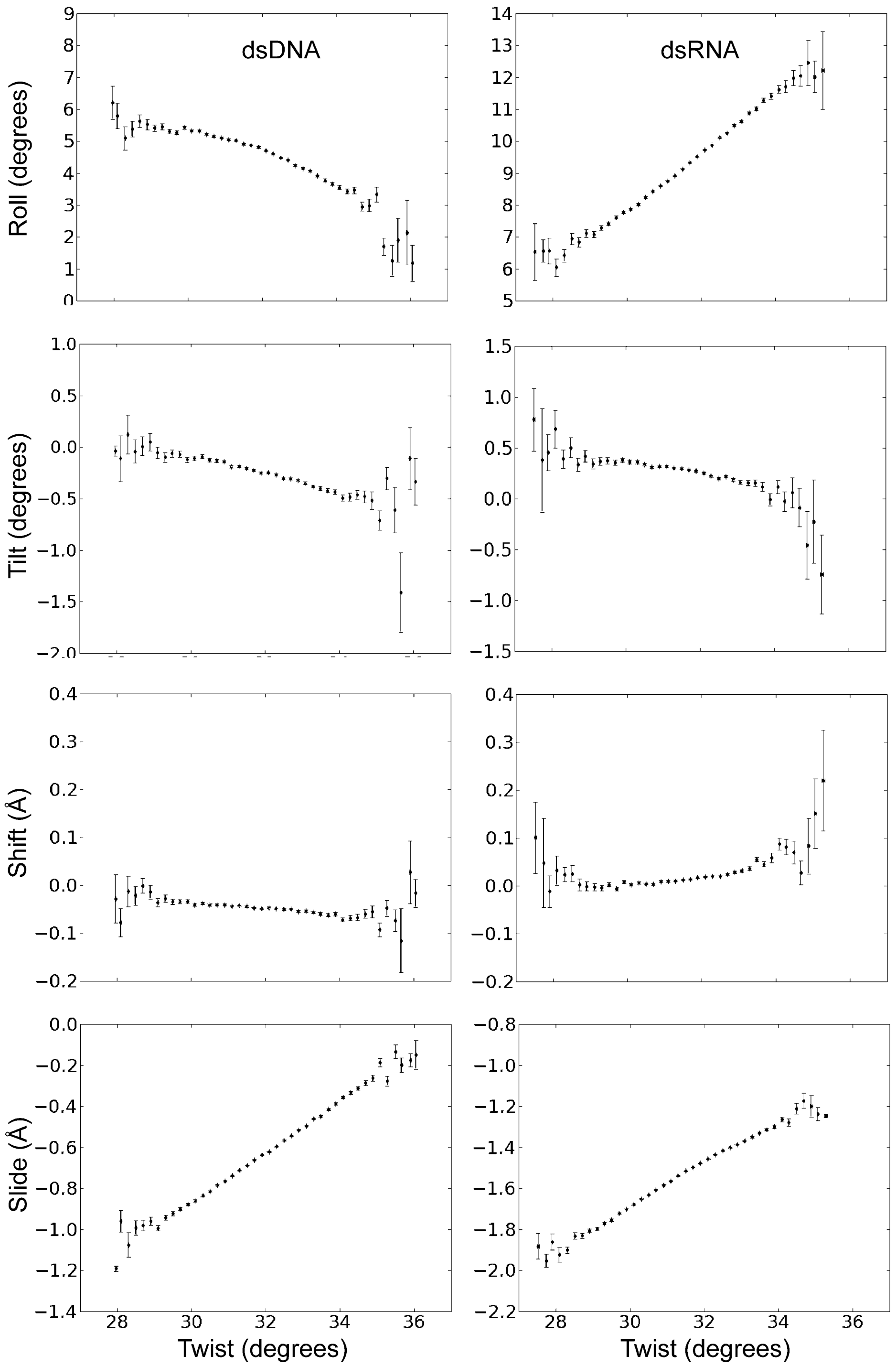


**Figure S3.** Correlation of local base pair step parameters with helical twist of DNA (left column of plots) and RNA (right column of plots). The plots were generated from the analysis of a total of ~100000 regularily spaced snapshots taken during ~1 μs unrestraint simulations. The data was analysed in twist intervals of 0.2° (x-axis) calculating the mean helical twist and the mean selected helical coordinate within the interval. Errors (shown as bars) were calculated as mean standard errors within each intervall.

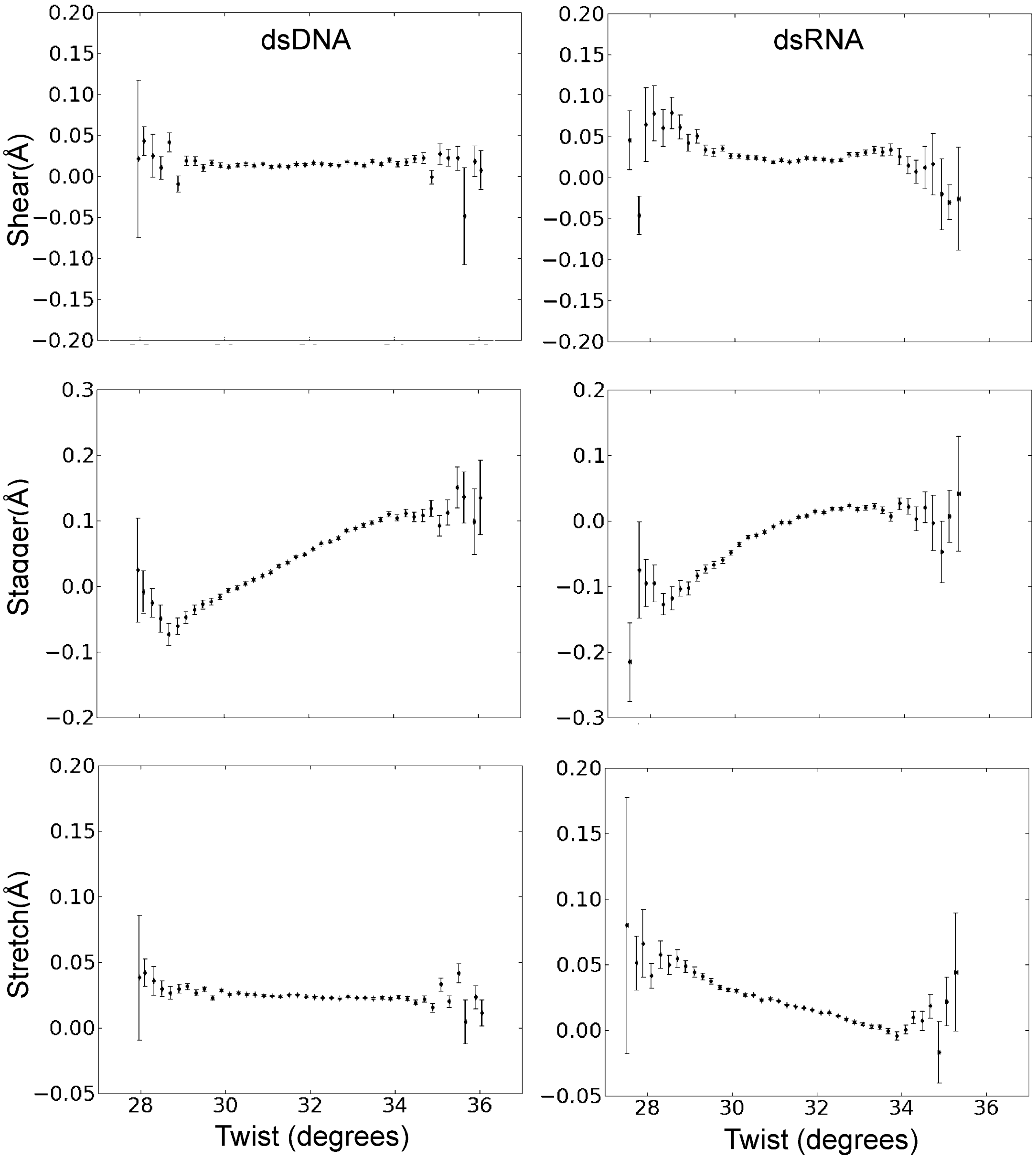


**Figure S4.** Correlation of translational local base pair parameters with helical twist of DNA (left column of plots) and RNA (right column of plots). The plots were generated from the analysis of a total of ~100000 regularily spaced snapshots taken during ~1 μs unrestraint simulations. The data was analysed in the same way as explained in the legend of Figure S1.

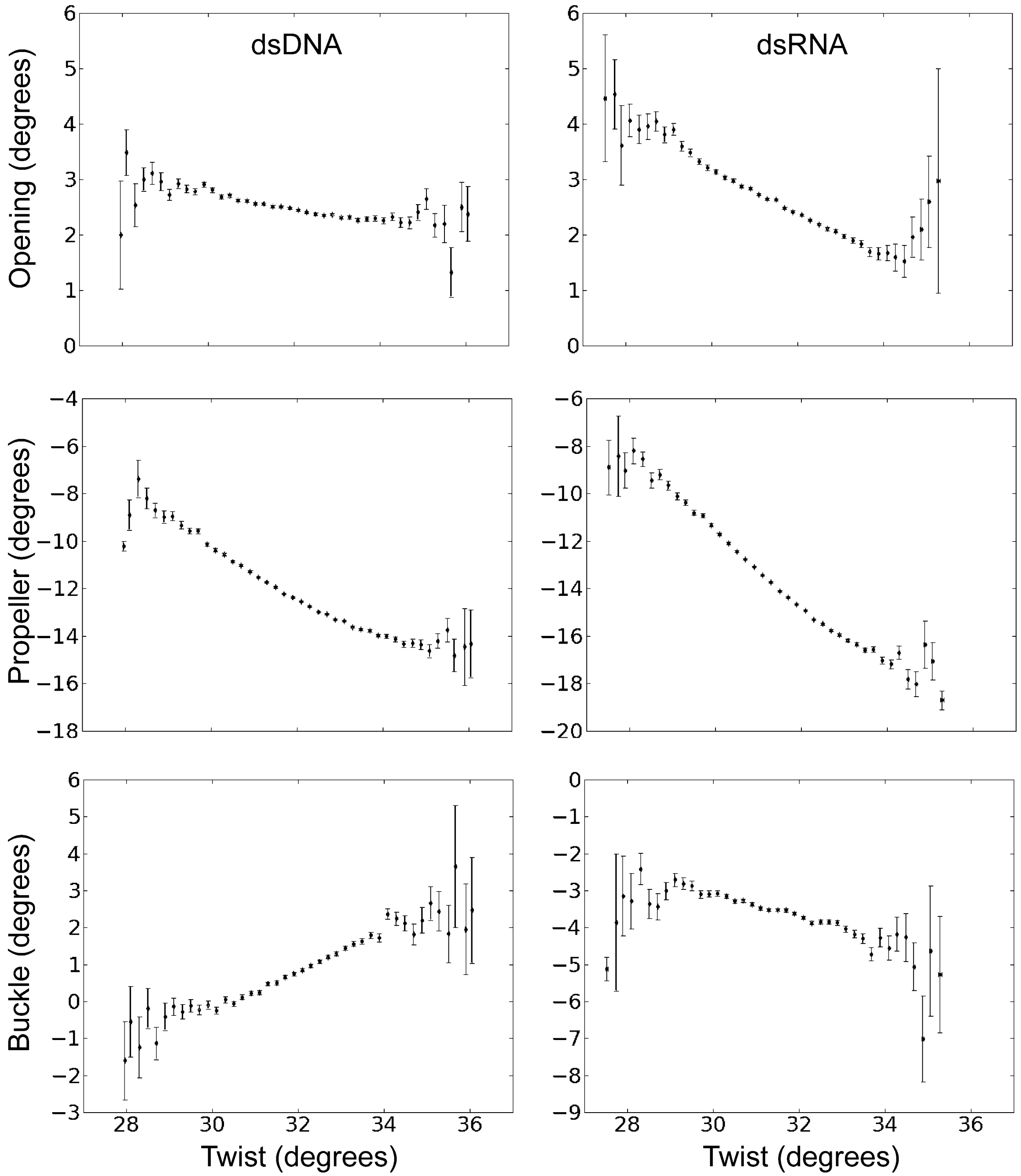


**Figure S5.** Correlation of angular local base pair parameters with helical twist of DNA (left column of plots) and RNA (right column of plots). The plots were generated from the analysis of a total of 100000 regularily spaced snapshots taken during 1 μs unrestraint simulations. The data was analysed in same way as explained in the legend of Figure S1.

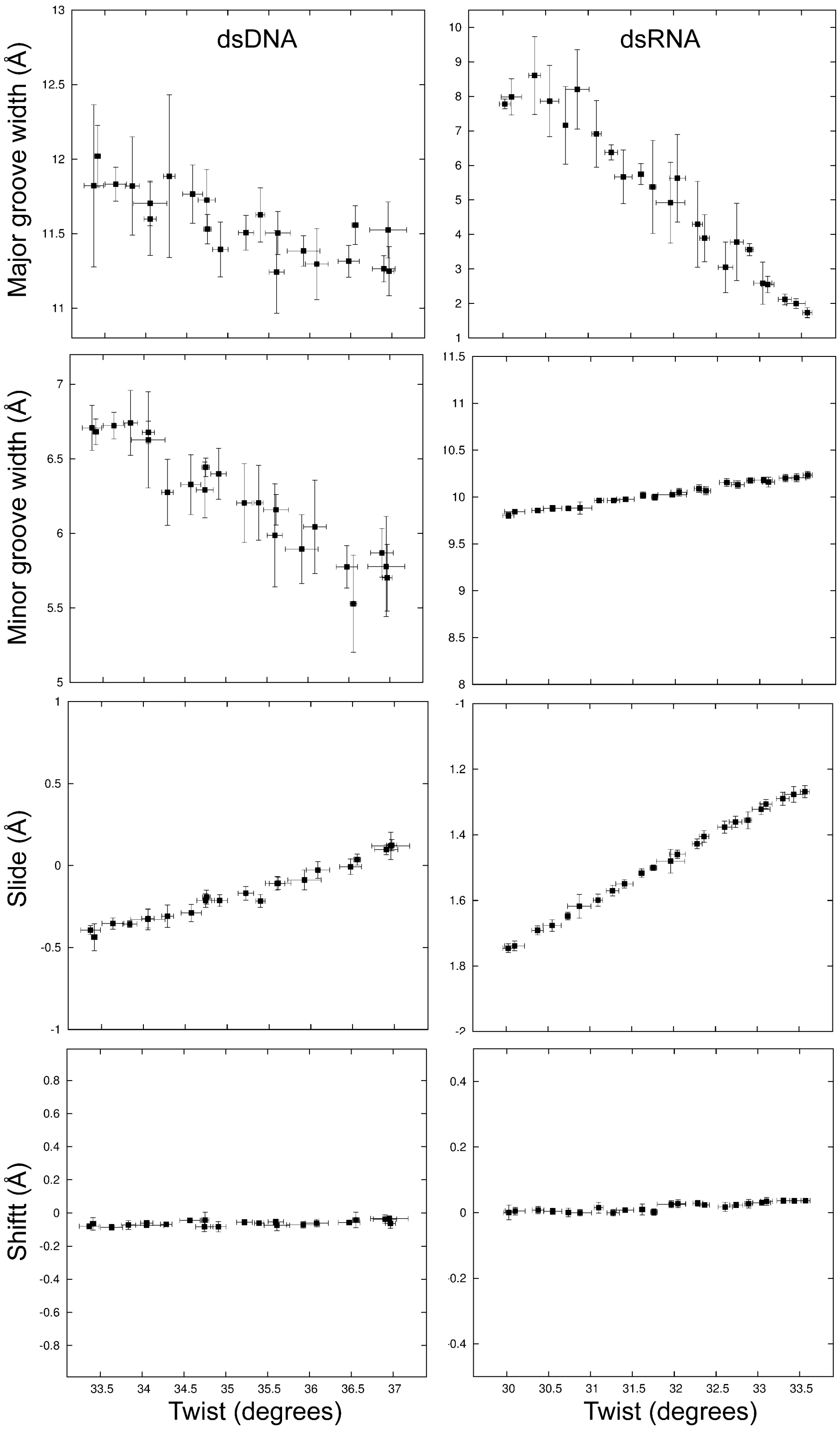


**Figure S6.** Variation of major and minor groove width, slide and shift during simulations with a torque restraint on the total twist of the DNA (left panels) and RNA (right panels). The mean twist per base pair step was changed in steps of ~0.15°. Error bars were calculated by splitting the recorded data for each twist restaining simulation into 5 intervals and calculating the standard variation over these intervals.

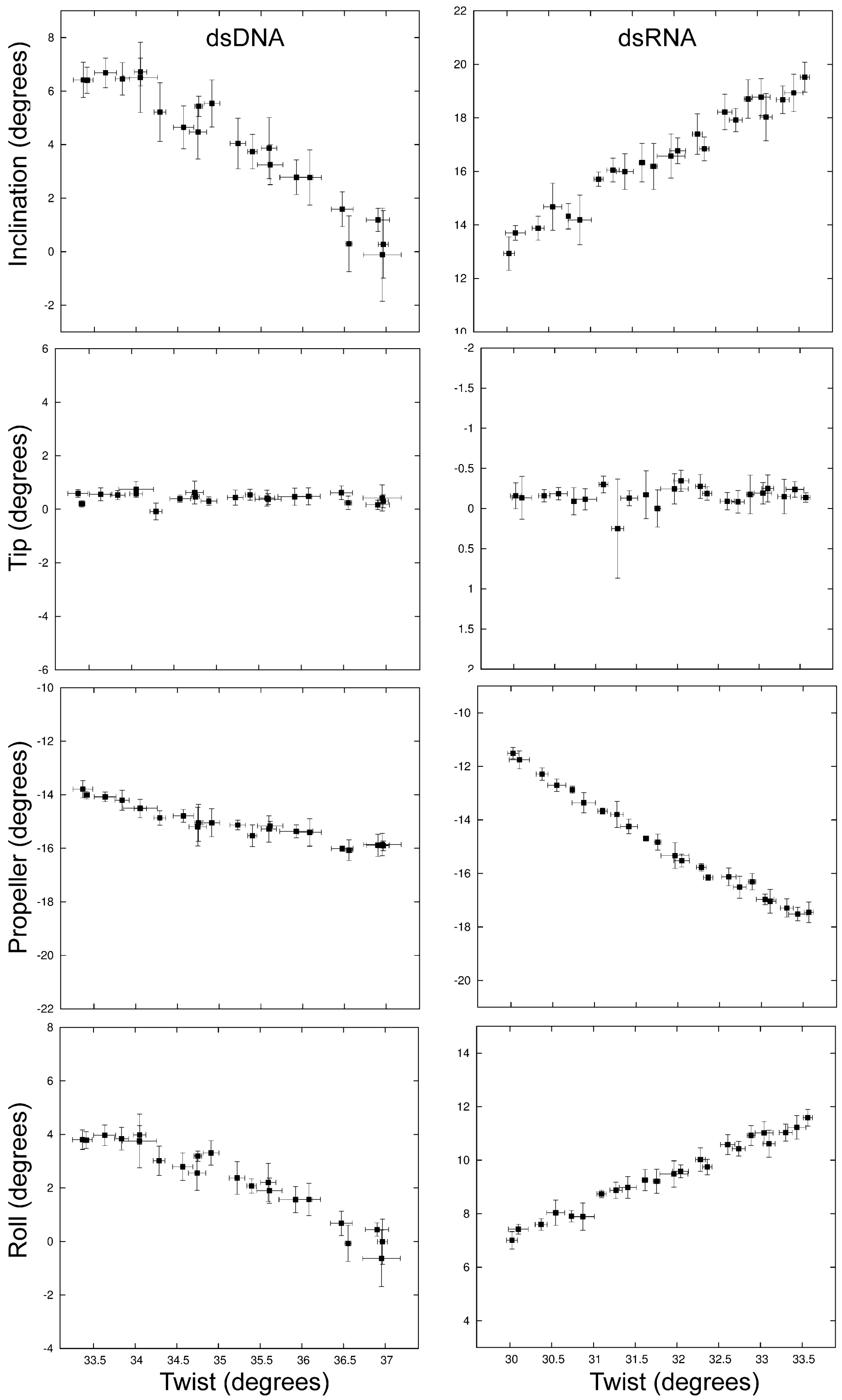


**Figure S7.** Variation of selected angular helical parameters inclination and tip, roll and propeller during simulations with a torque restraint on the total twist of the DNA (left panels) and RNA (right panels). The mean twist per base pair step was changed in steps of ~0.15°. Error bars were calculated by splitting the recorded data for each twist rrestaining simulation into 5 intervals and calculating the standard variation over these intervals.
